# A reduction in malaria transmission intensity in Northern Ghana after 7 years of indoor residual spraying

**DOI:** 10.1186/s12936-017-1971-0

**Published:** 2017-08-10

**Authors:** Sylvester Coleman, Samuel K. Dadzie, Aklilu Seyoum, Yemane Yihdego, Peter Mumba, Dereje Dengela, Philip Ricks, Kristen George, Christen Fornadel, Daniel Szumlas, Paul Psychas, Jacob Williams, Maxwell A. Appawu, Daniel A. Boakye

**Affiliations:** 1 USAID President’s Malaria Initiative Africa Indoor Residual Spraying Project, Accra, Ghana; 2 USAID President’s Malaria Initiative Africa Indoor Residual Spraying Project, Abt Associates Inc, 4550 Montgomery Ave, Suite 800 N, Bethesda, MD 20814 USA; 3 Noguchi Memorial Institute for Medical Research, University of Ghana, Legon, Ghana; 4 President’s Malaria Initiative/Malaria Branch, Centers for Disease Control and Prevention, Atlanta, GA USA; 5 President’s Malaria Initiative/U.S. Agency for International Development, 1300 Pennsylvania Avenue NW, Washington, DC USA; 6 Armed Forces Pest Management Board, 172 Forney Road, Forest Glen Annex, Silver Spring, MD 20910 USA; 7 University of Florida Emerging Pathogens Institute, Gainesville, FL USA; 8 The Johns Hopkins University, Washington, D.C. Metro Area USA

**Keywords:** Indoor residual spraying, Malaria transmission indicators, Parity rates, Entomological inoculation rate, Northern Ghana

## Abstract

**Background:**

Indoor residual spraying (IRS) is being implemented as one of the malaria prevention methods in the Northern Region of Ghana. Changes in longevity, sporozoite and entomological inoculation rates (EIRs) of major malaria vectors were monitored to assess the impact of IRS in selected districts.

**Methods:**

Monthly human landing catches (HLCs) were used to collect mosquitoes from sentinel sites in three adjacent districts between July 2009 and December 2014: Savelugu Nanton (SND) where IRS had been implemented from 2008 to 2014; Tolon Kumbungu (TKD) where IRS had been implemented between 2008 and 2012 and Tamale Metropolis (TML) with no history of IRS. Mosquitoes were morphologically identified to species level and into sibling species, using PCR. Samples of *Anopheles gambiae* sensu lato (s.l.) were examined for parity and infectivity. EIR was calculated from biting and infectivity rates of malaria vectors.

**Results:**

Parity rates of *An. gambiae* s.l. decreased significantly (p < 0.0001) in SND from 44.8% in 2011 to 28.1% by 2014, and in TKD from 53.3% in 2011 to 46.6% in 2012 (p = 0.001). However 2 years after IRS was discontinued in TKD, the proportion of parous *An. gambiae* s.l. increased significantly to 68.5% in 2014 (p < 0.0001). Parity rates in the unsprayed district remained high throughout the study period, ranging between 68.6% in 2011 and 72.3% in 2014. The sum of monthly EIRs post-IRS season (July–December) in SND ranged between 2.1 and 6.3 infective bites/person/season (ib/p/s) during the 3 years that the district was sprayed with alphacypermethrin. EIR in SND was reduced to undetectable levels when the insecticide was switched to pirimiphos methyl CS in 2013 and 2014. Two years after IRS was withdrawn from TKD the sum of monthly EIRs (July–December) increased by about fourfold from 41.8 ib/p/s in 2012 to 154.4 ib/p/s in 2014. The EIR in the control area, TML, ranged between 35 ib/p/s in 2009 to 104.71 ib/p/s by 2014.

**Conclusions:**

This study demonstrates that IRS application did have a significant impact on entomological indicators of malaria transmission in the IRS project districts of Northern Ghana. Transmission indicators increased following the withdrawal of IRS from Tolon Kumbungu District.

## Background

The use of indoor residual spraying (IRS) and insecticide-treated nets (ITNs) as key vector control interventions has seen a massive scale-up over the last decade [1]. This scale-up together with other case management and preventive measures were reported to have contributed to a global decline in malaria mortality rates by about 47% between 2000 and 2013, and by 54% in the World Health Organization (WHO) African Region [2]. It is known that IRS as a vector control intervention can be highly effective when over 80% of the target population are covered [3]. The effectiveness of IRS has been demonstrated through surveys that show a reduction in entomological indices of transmission [4–6] or significant reduction in malaria prevalence [4], morbidity and mortality [7, 8]. However several challenges threaten the efficacy of IRS. Studies have found that the scale up of IRS and ITNs contribute to changes in vector behaviour [5, 9], and changes in vector species composition or selection for certain traits that may support rapid evolution of insecticide resistance [10]. In areas where pyrethroid resistance is widespread, non-pyrethroid insecticides can be used to maintain the efficacy of IRS. However, new insecticides and formulations tend to be more expensive than the previously used pyrethroids, and have partly contributed to the scale-down of IRS coverage in some countries, including Ghana [2, 11].

In Ghana, IRS forms a key component of the national malaria control strategic plan to reduce the malaria burden in the country [12, 13]. Currently, IRS is being implemented in the Northern Region through support from the United States President’s Malaria Initiative (PMI) and in Upper West Region and Obuasi municipality in the Ashanti region supported by the Global Fund. The PMI supported IRS programme in northern Ghana was scaled down from 9 districts in 2012 to 4 districts in 2013 and 2014, as a result of the switch of insecticides from pyrethroid to a more expensive organophosphate. However the national malaria control strategic plan calls for national scale-up of IRS in the country. In view of the sub-national differences in intensity of malaria transmission [14, 15] and the logistical difficulties of IRS implementation in some areas, there is the need to document the epidemiological and entomological impact of already existing IRS programmes in the different ecological zones of the country [15] before a rapid country-wide scale-up of this intervention. This is needed to provide evidence for decision-making and guide future programme implementation.

Findings of the impact of the IRS operations, as well as IRS withdrawal on the sporozoite and entomological inoculation rates across two sentinel districts of the PMI IRS program in Northern Region, Ghana are discussed in comparison with an unsprayed district. The impact of IRS on parity rates as an indicator of longevity was also measured in sprayed and unsprayed districts.

## Methods

### Study areas

**Fig. 1:**
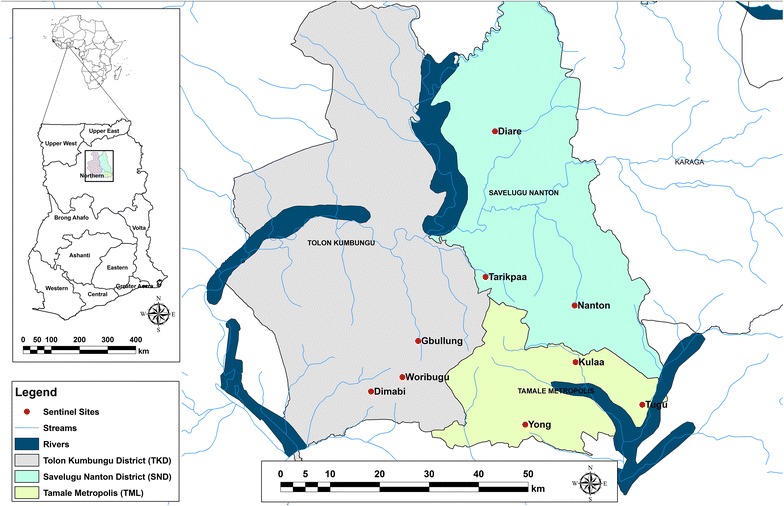
Map of study area located within the Northern region of Ghana

Three districts in the IRS programme area were selected for entomological monitoring, namely: Savelugu Nanton District (hereafter SND) (9.25° and 10.8°N and 0.33° and 1.00°W), Tolon Kumbungu District (TKD) (9.15° and 10.70°N, 0.52° and 1.23°W), and Tamale Metropolis (TML) (9.16° and 9.34° N and 00.34° and 00.59^o^ W). Three rural communities within each district were used as sentinel sites (Fig. 1). Tarikpaa, Diare and Nanton communities under SND and Gbullung, Woribugu and Dimabi communities under TKD were purposively selected as entomological sentinel sites. Three rural communities (Kulaa, Tugu and Yong) under the Tamale metropolis with no history of IRS and no near term plans for IRS implementation were selected as control sentinel sites for comparison of trends on longevity, sporozoite infectivity and entomological inoculation rates.

All the study sites are rural and located in Ghana’s northern savannah zone, which experiences a unimodal rainy season that occurs between May and November, with the peak months (July to September) receiving between 150 and 250 mm of rainfall per month. The rainy season is followed by a dry season that lasts from December until March [16]. The annual range of temperature is between 25 and 30 °C [16], which appears to be favourable for *Anopheles* larval development. A typical housing complex found in the study area consists mainly of compounds made up of round huts roofed with thatch and scattered over large farmlands.

Mean daily rainfall data from the study area was obtained from weather stations of the Ghana Meteorological Services Department in Savelugu and Tamale, and Savannah Agricultural Research Institute (SARI)—Tolon Kumbungu.

### IRS campaigns and insecticides used

IRS was conducted once annually, timed to coincide with the first or second month of the rainy season depending on the year. SND and TKD started IRS in 2008, beginning with the inexpensive pyrethroid insecticide alphacypermethrin (Fendona 5 WP) at 30 mg/m^2^ in 2008 and 2009 which has a residual life of 4–6 months. In 2010 both districts were sprayed with the alternative pyrethroid, deltamethrin (Pali 25 WG) with a residual life of 3–6 months, at a rate of 20 mg/m^2^. Both districts reverted to alphacypermethrin in 2011. In 2012, the two district programmes diverged. Due to emerging insecticide resistance concerns [17], communities in SND were sprayed with an organophosphate insecticide (pirimiphos methyl CS formulation at a rate of 1 g/m^2^) with a residual life of 4–6 months, whereas communities in TKD were still sprayed with alphacypermethrin. IRS was discontinued in TKD in 2013 due largely to cost considerations, but continued in SND with pirimiphos methyl CS through 2014. Meanwhile the neighbouring control district TML received no IRS throughout. The timeframe for IRS rounds is presented in Table 1.

**Table 1:** IRS implementation schedule and insecticide usage for the PMI IRS programme, Northern Ghana 2008–2014

Districts	Insecticide used and spray coverage (%)							
Calendar year:	Pre-2008	2008	2009	2010	2011	2012	2013	2014
IRS year:		1	2	3	4	5	6	7
Savelugu-Nanton District (SND)	No IRS	Alphacypermethrin (88.4%)	Alphacypermethrin (93.6%)	Deltamethrin (98.7%*)*	Alphacypermethrin (87.5%)	Pirimiphos-methyl CS (89.7%)	Pirimiphos-methyl CS (91.1%)	Pirimiphos-methyl CS (68.0%)
Tolon-Kumbungu District (TKD)	No IRS	Alphacypermethrin (91.3%)	Alphacypermethrin (93.4%)	Deltamethrin (98.0%*)*	Alphacypermethrin (91.1%)	Alphacypermethrin (91.9%)	No IRS	No IRS
Tamale Metropolis (TML)	No IRS	No IRS	No IRS	No IRS	No IRS	No IRS	No IRS	No IRS
*Entomological monitoring*								
All districts	None	Periodic	Monthly (post-IRS)	Monthly	Monthly	Monthly	Monthly	Monthly

Percentages in parenthesis = yearly IRS coverage of sprayable houses

### Spray coverage and quality

The programme implemented intensive mobilization and sensitization campaigns. Spray operators were also well trained and closely supervised to make sure the required quality of operation was achieved [18, 19].

### ITN campaigns, coverage and use

Data on ITN coverage and use was obtained at the regional level from the 2008 and 2014 Demographic and Health Surveys and the 2011 Multiple Indicator Cluster Survey [15, 20, 21], each of which were conducted nationwide at the end of the rainy season.

### Adult mosquito collections

Using the human landing catch (HLC) method [22] monthly mosquito collections were conducted simultaneously across all study sites. In each sentinel community, 8 trained mosquito collectors worked in two teams of four, working in 2 houses each night from 6 p.m. to 6 a.m. In each house 2 collectors worked indoors whilst the other 2 worked outdoors. Collections were done for a total of 4 nights to sample 8 houses in the community per month (24 houses in a district per month). Collectors shifted between indoor and outdoor every hour and were allowed to take 10 min breaks between shifts. Due to logistical challenges, there were occasional gaps in monthly monitoring, but a total of 61 out of 65 months were covered.

### Mosquito identification and processing

#### Vector species identification

Mosquitoes collected were morphologically identified to species level using taxonomic keys [23]. Mosquito samples of the *Anopheles gambiae* complex were further identified into sibling species using ribosomal DNA-polymerase chain reaction (PCR) [24] and into molecular forms following the PCR–RFLP (restriction fragment length polymorphism) procedure [25].

#### Parity dissections

About 30% of unfed *An. gambiae* s.l. identified morphologically was dissected per site per month, except for months when very few numbers of *An. gambiae* s.l. females were captured. Three of every ten unfed *An. gambiae* s.l. identified morphologically from the hourly collections per site were selected for dissection. The selected mosquitoes were dissected and their ovaries examined for parity by observing the degree of coiling in the ovarian tracheoles [26]. Due to financial and logistical challenges, monthly parity data are available from 2011 to 2014.

#### Circumsporozoite assay

The head and thoraces of a subset (~10%) of *Anopheles* mosquitoes collected were tested using enzyme-linked immunosorbent assay (ELISA) [27] for the presence of circumsporozoite protein (CSP) of *Plasmodium falciparum*, the major malaria parasite in the study areas. These tests were performed at the Noguchi Memorial Institute for Medical Research (NMIMR) laboratory in Accra, Ghana.

### Data analysis

The number of mosquitoes collected per month which belonged to important *Anopheles* vector species (*An. gambiae* s.l. and *An. funestus* group) was used as the denominator to calculate the following ratios:

- Parity rate
*The proportion of parous mosquitoes among unfed and blood meal seeking mosquitoes. Parity was used as a proxy measure for the daily survival rate and average life span of the vector population*
- Monthly sporozoite rate
*Number of mosquitoes found positive for the presence of circumsporozoite proteins/number of mosquitoes tested*
- The entomological inoculation rates (EIRs) were calculated by the formula
  $$\begin{aligned} EIR{\text{-}}daily &= Daily\;human\;biting\;rates \\ & \quad \times sporozoite\;rate \end{aligned}$$
  $$\begin{aligned} Post \, IRS \, EIRs = \sum \, Monthly \, IRS \, EIRs \, (July{-}December) \end{aligned}$$

Yearly variations in parity and sporozoite rate for *Anopheles* spp. collected from IRS and unsprayed districts were compared through a z test for differences in proportions. Pearson’s correlation was used to assess the relationship between rainfall and EIRs. All tests were performed at 0.05 significance level, using SPSS version 20 and Microsoft Excel^®^.

## Results

### Spray coverage

The timeframe for IRS implementation, insecticide used and yearly coverage is presented in Table 1. The intensive mobilization and sensitization campaigns implemented by the programme ensured high spray coverage (Table 1). Over 80–90% coverage was achieved in both districts each year, with the exception of 68% coverage in SND in 2014 [19], attributed to low acceptance among certain town populations.

### ITN campaigns, coverage and use

ITN coverage and use was found to be high across all study areas. This reflected the large-scale efforts of the National Malaria Control Programme (NMCP) and international partners since 2006 to promote the ownership and use of ITNs in Northern Region. ITNs were distributed largely through mass door-to-door ITN distribution campaigns in 2010 and 2012, then through school and health facility distributions in 2013–2014 (Fig. 3) [13, 17]. ITN ownership in Northern Region increased from 54% in the 2008 Demographic and Health Survey to about 71% by the 2014 DHS survey, with an average of 1.7 ITNs per household in 2014 [20, 21]. ITN usage among children under age 5 and pregnant women were 58% and 61%, respectively, in 2014 [21].

#### Anopheles species composition and relative abundance

**Fig. 2:**
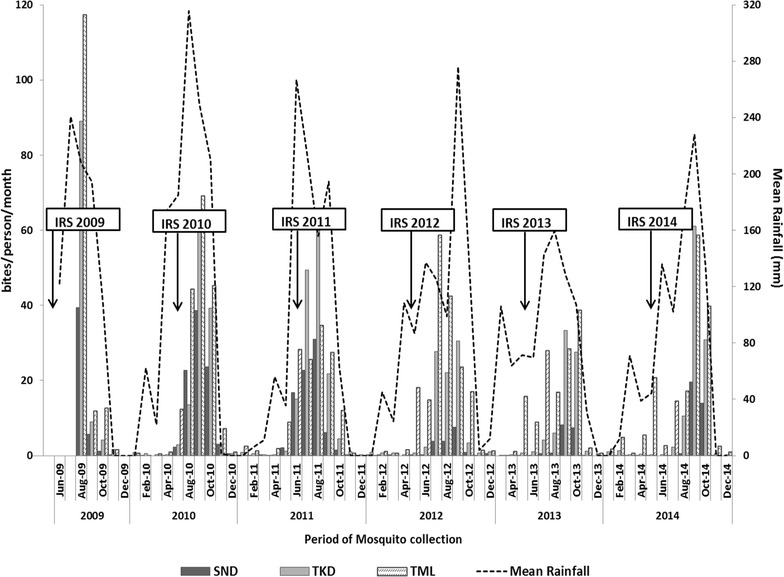
Biting rates of *An. gambiae* s.l. collected from sentinel sites between August 2009 and December 2014

A total of 192,259 adult female *Anopheles* mosquitoes belonging to 5 different species were collected over the 6 years (2009–2014). *An. gambiae* s.l. (95.3%) was the most abundant species, followed by *An. nili* (2.4%) and *An. funestus* (1.7%). *An. rufipes* and *An. pharoensis* made up 0.2 and 0.4% respectively of the total *Anopheles* mosquitoes collected (Table 2). There was monthly and yearly variation in abundance of the main vectors (*An. gambiae* s.l. and *An. funestus*) in all the areas. The vector abundance in all the areas coincided with the peak of the rainy season (Fig. 2). The mean biting rates of vectors in the unsprayed communities were higher than biting rates for the IRS areas across all the years. The biting rate of the main vectors from all sites significantly reduced over the duration of the study (2009–2014).

**Table 2:** Total number of *Anopheles* caught per study site

*Anopheles* species collected	Savelugu-Nanton District (SND)	Tolon-Kumbungu District (TKD)	Tamale metropolis (TML)	Total
*An. gambiae* s.l.	27,648 (98.9%)	63,500 (95.6%)	92,111 (94.1%)	183,259 (95.3%)
*An. funestus*	76 (0.3%)	1849 (2.8%)	1378 (1.4%)	3303 (1.7%)
*An. rufipes*	42 (0.2%)	117 (0.2%)	259 (0.3%)	418 (0.2%)
*An. nili*	58 (0.2%)	854 (1.3%)	3632 (3.7%)	4544 (2.4%)
*An. pharoensis*	140 (0.5%	111 (0.2%)	484 (0.5%)	735 (0.4%)
	27,964	66,431	97,864	192,259

PCR analysis on *An. gambiae* s.l. collected between 2009 and 2013 showed that *An. coluzzii* and *An. gambiae* sensu stricto (s.s.) were present in all the sites in varying proportions with *An. gambiae* s.s. predominating (75–89%) between 2009 and 2013. However, in 2014 *An. coluzzii* was the predominant molecular form making up over 91% of the *An. gambiae* species from all sites (Table 3). Five *An. arabiensis* were identified out of 348 *An. gambiae* s.l. analysed in 2014.

**Table 3:** Distribution of molecular forms of *An. gambiae* s.l. caught per study site

Year	Total # samples analysed	Savelugu-Nanton District (SND)		Tolon-Kumbungu District (TKD)		Tamale Municipality (TML)		
		*An. gambiae*	*An. coluzzii*	*An. gambiae*	*An. coluzzii*	*An. gambiae*	*An. coluzzii*	M/S hybrid
2009	200	49	0	58	17	69	7	0
2010	312	68	12	96	16	98	22	0
2011	135	46	4	33	8	39	5	0
2012	80	18	8	25	5	18	6	0
2013	161	54	0	43	13	44	7	0
2014	263	0	58	10	80	16	97	2

#### Parity rates of vectors

**Fig. 3:**
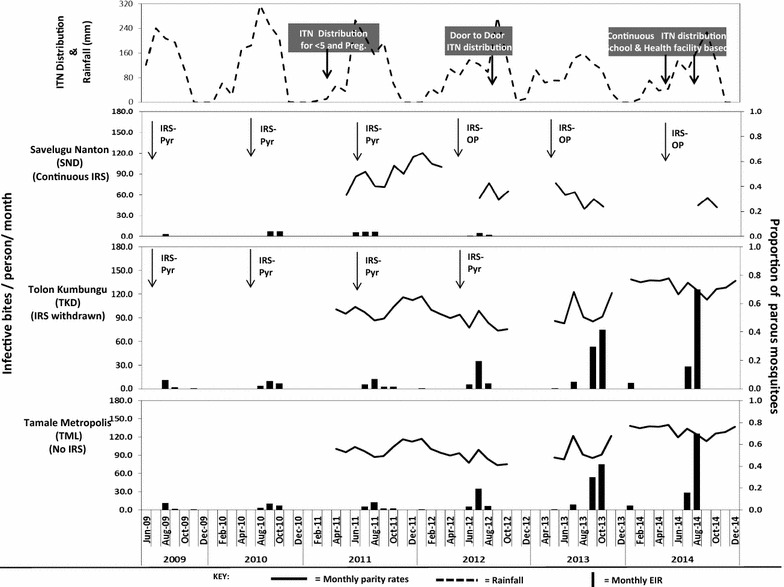
Proportion of parous *An. gambiae* s.l. and entomological inoculation rate from IRS and non-IRS districts. Parity data is not available for 2009 and 2010

Higher proportions of parous female *An. gambiae* s.l. were collected from the unsprayed district compared to sprayed districts (Table 4; Fig. 3) (p < 0.0001). Whereas a general decrease in the proportion of parous mosquitoes was observed across the IRS sites, parity rates in the IRS withdrawn and the unsprayed district remained high. The proportion of parous *An. gambiae* s.l. collected in SND in 2011 was reduced from 44.8 to 37.4% in 2012 when the district was sprayed with pirimiphos methyl CS (z = 2.55, p = 0.011). The proportion of parous females was further reduced to 27.5% (z = 3.22, p = 0.001) in 2013. Despite a significant reduction in parity rates in 2012 (z = 3.24, p = 0.001) when IRS was being implemented, the withdrawal of IRS in 2013 from TKD resulted in an increase in the proportion of parous (older) mosquitoes from 46.6% in 2012 to 50.4% in 2013 (z = −2.07, p = 0.038). Parity rates in TKD increased to 68.5% in 2014 (z = −9.79, p < 0.0001). Parity rates in TML remained high, increased somewhat from 64.3% recorded in 2013 to 72.3% in 2014 (z = −6.11, p < 0.0001) (Table 4).

**Table 4:** A comparison of parous *An. gambiae* s.l. collected from IRS and non-IRS districts between 2011 and 2014

Study district/year compared	Compared parity rate (%)	% Change	Pooled sample proportion	Standard error	Z test statistic	p value	Remark
Savelugu Nanton District (SND)							
2011–2012	44.8 → 37.4%	−16.5	0.421	0.029	2.549	0.011*	Change from ACP to PM
2012–2013	37.4 → 27.5%	−26.5	0.324	0.031	3.222	0.001*	Sprayed with PM
2013–2014	27.5 → 28.1%	+2.1	0.277	0.034	−0.168	0.867	Sprayed with PM
Tolon Kumbungu District (TKD)							
2011–2012	53.3 → 46.6%	−12.6	0.499	0.021	3.242	0.001*	IRS continued with ACP
2012–2013	46.6 → 50.4%	+8.0	0.490	0.018	−2.071	0.038	IRS withdrawn
2013–2014	50.4 → 68.5%	+36.0	0.567	0.019	−9.793	0.000*	IRS withdrawn
Tamale Metropolis (TML)							
2011–2012	68.6 → 65.8%	−4.1	0.671	0.016	1.730	0.084	No IRS
2012–2013	65.8 → 64.3%	−2.2	0.649	0.015	0.991	0.322	No IRS
2013–2014	64.3 → 72.3%	+12.5	0.681	0.013	−6.113	0.000*	No IRS

*ACP* alpha-cypermethrin, *PM* pirimiphos-methyl

* p value significant at 0.05 significance level; −ve% represents percentage reduction; +ve% represents percentage increase

#### Sporozoite rate of local vector species

Whereas sporozoite infection in SND was reduced to a level that could not be detected in 2013 and 2014, the sporozoite rates in TKD (after IRS withdrawal) and Tamale (unsprayed district) remained high (p < 0.05) (Table 5) in comparison to the sprayed area.

**Table 5:** Entomological parameters of malaria transmission recorded for vector species from all district

District and transmission indicators	2009	2010	2011	2012	2013	2014
Savelugu Nanton District (SND)						
Insecticide sprayed	PY	PY	PY	OP	OP	OP
Parity rate (# dissected)	–	–	44.8% (806)	37.4% (457)	27.5% (469)	28.1% (285)
Sporozoite rates (# examined)	0.4% (550)	0.2% (882)	0.4% (794)	1.9% (530)	0.0% (841)	0.0% (400)
∑ Monthly EIRs post-IRS season (July–December)	2.1	6.9	6.3	3.5	0.0	0.0
Tolon Kumbungu District (TKD)						
Insecticide sprayed	PY	PY	PY	PY	No IRS	No IRS
Parity rate (# dissected)	–	–	53.3% (1140)	46.6% (1203)	50.4% (2066)	68.5% (1089)
Sporozoite rates (# examined)	0.5% (582)	0.5% (1229)	0.5% (1453)	3.6% (1048)	3.3% (883)	4.9% (388)
∑ Monthly EIRs post IRS season (July–December)	13.8	21.0	24.0	41.8	137.9	154.4
Tamale Metropolis (No IRS)						
Insecticide sprayed	No IRS	No IRS	No IRS	No IRS	No IRS	No IRS
Parity rate (# dissected)	–	–	68.6% (1625)	65.8% (1764)	64.3% (2687)	72.3% (2395)
Sporozoite rates (# examined)	1.6% (939)	2.0% (1764)	3.2% (1307)	1.9% (1275)	3.1% (1140)	2.2% (1754)
∑ Monthly EIRs post-IRS season (July–December)	35.0	109.4	103.1	125.6	188.0	104.7
Mean annual rainfall (mm)	149.6	120.0	87.4	87.9	77.3	79.9

No ovary dissections done in 2009 and 2010

Rainfall data obtained from Ghana Meteorological Services Dept. weather stations in Savelugu and Tamale and Savannah Agricultural Research Institute—Tolon Kumbungu

*PY* pyrethroids (alphacypermethrin: 2009, 2011 and 2012; deltamethrin 2010); *OP* organophosphate (pirimiphos methyl)

#### Entomological inoculation rate of local vector species

Malaria transmission was highly seasonal across all sites (Fig. 3), as expected. The highest EIRs were observed during the rainy season (May–October) across all the sites. Bivariate Pearson’s correlation analysis of the monthly EIRs with rainfall showed that the mean monthly EIRs had a positive correlation with rainfall across all the sentinel sites: SND (R = 0.447, p < 0.001), TKD (R = 0.276, p = 0.030) and TML (R = 0.282, p = 0.026). All infections in SND were detected during the rainy season. However, in TKD and TML malaria transmission still occurred in the dry season (Fig. 3).

The entomological inoculation rate (EIR), was lower in the IRS districts sprayed with the organophosphate compared to districts sprayed with pyrethroids, unsprayed and IRS withdrawn districts (Fig. 3). The EIRs during the post-IRS months (July–December) in the two IRS districts (SND and TKD) were suppressed until 2012 when the post-IRS EIR in TKD doubled from 24 infective bites/person/season (ib/p/s) in 2011 to 41.8 ib/p/s. A year-by-year comparison of the transmission intensity in TKD between July and December revealed that the sum of monthly EIRs during this period increased by about threefold in 2013 to 137.9 ib/p/s when IRS was discontinued and remained high in 2014 (Table 5). On the other hand, the sum of post-IRS monthly EIRs in SND was reduced to about half the levels in 2011 (i.e. from 6.3 ib/p/s in 2011 to 3.5 ib/p/s in 2012) just 1 year after the switch from pyrethroids to organophosphates. Transmission intensity was further reduced to a level that could not be detected by the sampling method used in 2013 and 2014 after the shift to organophosphate insecticide. There was yearly variation in EIRs in all the sites but EIRs in the control district (TML) remained high compared to the sprayed areas.

## Discussion

The major purpose of IRS is to reduce malaria transmission by reducing the survival of malaria vectors after entering and feeding on humans inside dwellings and consequently preventing transmission of malaria infection to others [28]. The general significant decline in the parity rates in the IRS district over the years (from 2011 to 2014) can likely be attributed to the effect of the IRS [5, 29]. Parity rates of vectors in TKD were reduced when IRS was being implemented in 2012 but increased steadily after the withdrawal of IRS in 2013 through 2014. Even though some climatic factors, such as temperature and humidity, are known to affect vector longevity and abundance of parous females [30], such climatic factors, as well as ITN ownership and use are expected to be similar across all IRS and non-IRS districts in this study, which are adjacent to each other. Therefore, the most likely factor for the low parity rate in the sprayed districts is the IRS operation in these districts, demonstrating the benefit of adding IRS where there is modest to high level ITN coverage.

The results confirms the seasonality of malaria transmission in all the study sites as indicated by previous studies in Northern Ghana [14, 31, 32] and justifies the one spray round per year approach adopted by the PMI IRS programme in the area.

The EIR in SND was low at the beginning of the study but increased subsequently in 2010 and 2011. The increase can likely be attributed to pyrethroid resistance in the area. However, the timely change to an organophosphate (pirimiphos methyl CS) resulted in reduction in EIR to undetectable levels in 2013 and 2014.

The study found *An. gambiae* s.l. to be the predominant vector species collected from all sentinel sites. This is consistent with studies in other parts of the country that also found *An. gambiae* s.l. to be the major *Anopheles* species feeding on humans in Ghana [14, 33]. The predominance of *An. gambiae* s.l. could in part be attributed to its high reproductive potential [34] and its high plasticity [35]. The relatively high number of *An. funestus* collected from TKD over the period could be attributed to the presence of rice irrigation farms in the area, as some studies [36, 37] have found that *An. funestus* prefers to breed in semi-permanent waters and sites shaded by vegetation, a condition provided by the irrigation sites in the area. The presence of the rice irrigation farms in the TKD area could have also created several breeding sites for mosquitoes even in the dry season and may account for the relatively high number of *An. gambiae* s.l. collected in TKD district.

Distribution of *An. coluzzii* and *An. gambiae* s.s. has been found to be dependent on ecological and geographical factors [38, 39]. Reduction in mean annual rainfall for the study area between 2009 and 2014 (from 149.6 mm in 2009 to 79.9 mm by 2014) could partly explain the general increase in *An. coluzzii* across all sites in 2014 compared to previous years. It is possible that temporary rainfall dependent larval habitats that could support breeding of *An. gambiae* s.s. [39] might have also declined over the period with the reduction in amount of rainfall (Table 5), thus restricting potential breeding sites to permanent irrigation fields that have been documented [38] to support breeding of *An. coluzzii*.

As a result of local factors such as irrigation, the EIR rates and entomological indices of transmission in TKD did not drop as low as observed in SND. The continuous use of pyrethroids for IRS in TKD in 2012 might have contributed to development of resistance to pyrethroids in 2012. By the end of 2012 *An. gambiae* s.l. had become highly resistant to alphacypermethrin. The 24 h mortality rates in WHO susceptibility tests conducted in 2012 was 76% compared to 97% recorded in 2011 [unpublished observations]. This in-part explains the increased malaria transmission intensity observed in TKD in 2012, bringing to the fore the important role that insecticide choice plays in the effectiveness of an IRS programme within the context of an insecticide resistance management strategy. Two years after the withdrawal of IRS malaria transmission intensity increased by about threefold in TKD, in spite of the high ITN coverage maintained in Northern Region by the door-to-door campaign in 2012 and the school-based campaigns in 2013 and 2014. This suggests that in the event that a decision is taken to discontinue IRS in a similar high burden savannah area, additional efforts such as intensive quality social and behaviour change communication (SBCC) to promote proper use of ITNs amongst all age groups and encourage correct malaria prevention and treatment behaviours at the household level may be warranted. Data from TML (unsprayed district) suggests strongly that IRS in the neighbouring district led to the reductions in transmission in the neighbouring district. The yearly fluctuations and relative reductions in transmission intensity in TML shows that factors other than IRS operational quality and pesticide choice, such as rainfall and ITN usage are going to affect transmission indices.

Central to the formulation of malaria control strategies is a more critical understanding of the relationship between malaria risk factors, EIRs and disease outcomes. Therefore collection and analysis of routine health information system data on how the reductions in the entomological indicators of malaria transmission translate to reduction in disease burden in the area would be important.

## Conclusions

The reduction in malaria transmission in the study districts demonstrates the effectiveness of IRS programmes and the potential benefits of their expansion. However, insecticide resistance and its resulting cost implications will determine impact and feasibility of IRS implementation.

The study demonstrated that the shift from the pyrethroid based IRS to organophosphates was effective in reducing the longevity of *Anopheles* vector species and eventually their malaria transmission potential. However, the withdrawal of IRS led to an increase in entomological indicators of malaria transmission, suggesting that maintenance of high local ITN coverage was not adequate to prevent a rebound of entomological indicators.

The study shows that if IRS is done optimally, it can contribute to significant reductions in key malaria transmission indices. Therefore collection and analysis of routine health information system data is important to complement entomological indicators of malaria transmission.

## References

[1] WHO (2015). World malaria report 2015.

[2] WHO (2014). World malaria report 2014.

[3] Rehman AM, Coleman M, Schwabe C, Baltazar G, Matias A, Gomes IR (2011). How much does malaria vector control quality matter: the epidemiological impact of holed nets and inadequate indoor residual spraying. PLoS ONE.

[4] Zhou G, Githeko AK, Minakawa N, Yan G (2010). Community-wide benefits of targeted indoor residual spray for malaria control in the Western Kenya Highland. Malar J.

[5] Aïkpon R, Sèzonlin M, Tokponon F, Okè M, Oussou O, Oké-Agbo F (2014). Good performances but short lasting efficacy of Actellic 50 EC indoor residual spraying (IRS) on malaria transmission in Benin, West Africa. Parasites Vectors.

[6] Akogbéto MC, Aïkpon RY, Azondékon R, Padonou GG, Ossè RA, Agossa FR (2015). Six years of experience in entomological surveillance of indoor residual spraying against malaria transmission in Benin: lessons learned, challenges and outlooks. Malar J.

[7] Bukirwa H, Yau V, Kigozi R, Filler S, Quick L, Lugemwa M (2009). Assessing the impact of indoor residual spraying on malaria morbidity using a sentinel site surveillance system in Western Uganda. Am J Trop Med Hyg.

[8] Chanda E, Coleman M, Kleinschmidt I, Hemingway J, Hamainza B, Masaninga F (2012). Impact assessment of malaria vector control using routine surveillance data in Zambia: implications for monitoring and evaluation. Malar J.

[9] Russell TL, Beebe NW, Cooper RD, Lobo NF, Burkot TR (2013). Successful malaria elimination strategies require interventions that target changing vector behavior. Malar J.

[10] Norris LC, Main BJ, Lee Y, Collier TC, Fofana A, Cornel AJ (2015). Adaptive Introgression in an African malaria mosquito coincident with the increased usage of insecticide-treated bed nets. Proc Natl Acad Sci USA.

[11] Chanda E, Mzilahowa T, Chipwanya J, Mulenga S, Ali D, Troell P (2015). Preventing malaria transmission by indoor residual spraying in Malawi: grappling with the challenge of uncertain sustainability. Malar J.

[12] The National Malaria Control Programme (NMCP) Ghana. Strategic plan for malaria control in Ghana 2014–2020.

[13] Gakpey K, Baffoe-Wilmot A, Malm K, Dadzie S, Bart-Plange C (2016). Strategies towards attainment of universal coverage of long lasting insecticide treated nets (LLINs) distribution: experiences and lessons from Ghana. Parasites Vectors.

[14] Appawu MA, Baffoe-Wilmot A, Afari EA, Dunyo S, Koram KA, Nkrumah FK (2001). Malaria vector studies in two ecological zones in southern Ghana. Afr Entomol.

[15] Ghana Statistical Service. Ghana Multiple Indicator Cluster Survey with an Enhanced Malaria Module and Biomarker, 2011, Final Report. Accra, Ghana.

[16] McSweeney C, New M, Lizcano G. UNDP climate change country profiles: Ghana. 2010. http://country-profiles.geog.ox.ac.uk/. Accessed 8 Aug 2016.

[17] The President’s Malaria Initiative (PMI). Ghana Malaria Operational Plan FY 2013.

[18] Research Triangle Institute (RTI) International. Indoor residual spraying (IRS) 2 for malaria control. Indefinite quantity contract (IQC) task order 1 final report, September 2009–February 2013. Research Triangle Park: Research Triangle Institute (RTI) International.

[19] President’s Malaria Initiative Malaria (PMI)|Africa IRS (AIRS) project indoor residual spraying (IRS 2) task order four. Ghana 2014 end of spray report. Bethesda: Abt Associates Inc.

[20] GSS, GHS, and ICF Macro (2008). Ghana demographic and health survey.

[21] Ghana demographic and health survey 2014: key indicators. GSS, GHS, and ICF Macro.

[22] WHO (2013). Malaria entomology and vector control guide for participants. Global Malaria Programme.

[23] Gillies MT, Coetzee M (1987). A supplement to the Anophelinae of Africa south of the Sahara (Afrotropical Region). Publ S Afr Inst Med Res.

[24] Scott JA, Brogdon WG, Collins FH (1993). Identification of single specimens of the *Anopheles gambiae* complex by the polymerase chain reaction. Am J Trop Med Hyg.

[25] Fanello C, Santolamazza F, Torre AD (2003). Simultaneous identification of species and molecular forms of the *Anopheles gambiae* complex by PCR-RFLP. Med Vet Entomol.

[26] Detinova TS (1962). Age grouping methods in Diptera of medical importance.

[27] Wirtz RA, Burkot TR, Graves PM, Andre RG (1987). Field evaluation of enzyme-linked immunosorbent assays for *Plasmodium falciparum* and *Plasmodium vivax* sporozoites in mosquitoes (Diptera: Culicidae) from Papua New Guinea. J Med Entomol.

[28] WHO. Indoor residual spraying: Use of indoor residual spraying for scaling up global malaria control and elimination. World Health Organization Global Malaria Programme. 2006. p. 11–2.

[29] Ossè R, Aïkpon R, Padonou GG, Oussou O, Yadouléton A, Akogbéto M (2012). Evaluation of the efficacy of bendiocarb in indoor residual spraying against pyrethroid resistant malaria vectors in Benin: results of the third campaign. Parasites Vectors.

[30] Ribeiro AL, Miyazaki RD, Rodrigues JS, Campelo Júnior JH (2013). Parity and influence of abiotic factors on Anopheles in the Manso dam, state of Mato Grosso, Brazil. Rev Soc Bras Med Trop.

[31] Appawu M, Owusu-Agyei S, Dadzie S, Asoala V, Anto F, Koram K (2004). Malaria transmission dynamics at a site in northern Ghana proposed for testing malaria vaccines. Trop Med Int Health.

[32] Kasasa S, Asoala V, Gosoniu L, Anto F, Adjuik M, Tindana C (2013). Spatio-temporal malaria transmission patterns in Navrongo demographic surveillance site, northern Ghana. Malar J.

[33] Yawson AE, McCall PJ, Wilson MD, Donnelly MJ (2004). Species abundance and insecticide resistance of *Anopheles gambiae* in areas of Ghana and Burkina Faso. Med Vet Entomol.

[34] Olayemi IK, Ande AT (2009). Life table analysis of *Anopheles gambiae* (Diptera: Culicidae) in relation to malaria transmission. J Vector Borne Dis.

[35] Sinka ME, Bangs MJ, Manguin S, Coetzee M, Mbogo CM, Hemingway J (2010). The dominant Anopheles vectors of human malaria in Africa, Europe and the Middle East: occurrence data, distribution maps and bionomic précis. Parasites Vectors.

[36] Dadzie S, Brenyah R, Appawu M (2013). Role of species composition in malaria transmission by the *Anopheles funestus* group (Diptera: Culicidae) in Ghana. J Vector Ecol.

[37] Gillies MT, De Million B (1968). The Anophelinae of Africa south of the Sahara.

[38] Wondji CS, Simard F, Fontenille D (2002). Evidence for genetic differentiation between the molecular forms M and S within the forest chromosomal form of *Anopheles gambiae* in an area of sympatry. Insect Mol Biol.

[39] Diabate A, Baldet T, Chandre C, Dabire KR, Kengne P, Simard F (2003). Kdr mutation, a genetic marker to assess events of introgression between the molecular M and S forms of *Anopheles gambiae* (Diptera: Culicidae) in the tropical savannah area of West Africa. J Med Entomol.

